# Reliability and validity of the Japanese version of the active-emphatic listening scale

**DOI:** 10.1186/s40359-020-00422-4

**Published:** 2020-06-11

**Authors:** Keigo Asai, Taku Hiraizumi, Reino Hanzawa

**Affiliations:** 1 Graduate School of Education, Hokkaido University of Education, 1-15-55, Shiroyama, Kushiro-shi, Hokkaido 085-8580 Japan; 2 Faculty of General Welfare, Tohoku Fukushi University, 1-8-1 Kunimi, Aoba-ku, Sendai, Miyagi 981-8522 Japan

**Keywords:** Active-empathic listening scale (AELS), Active listening, Empathy, Japanese version, Test reliability, Test validity

## Abstract

**Background:**

Active-emphatic listening is active listening that focuses on empathy. The Active-Empathic Listening Scale (AELS) is a self-report scale comprising three dimensions: sensing, processing, and responding. However, translated versions are not available for languages used in Asian countries, such as Japanese. The aim of the present study was to demonstrate and report on the reliability and validity of the Japanese version of the AELS.

**Methods:**

After the scale was back translated, 728 Japanese university students completed the Japanese AELS. Of those participants, 566 responded to Japanese versions of the Interpersonal Reactivity Index (IRI) and Encode, Decode, Control, and Regulate Model (ENDCOREs) for validation, and the Japanese AELS was administered again to 59 participants after 3 weeks, to determine test-retest reliability. This study used confirmatory factor analysis to validate the scale’s three-factor structure. To evaluate reliability, Cronbach’s α, McDonald’s omega, and intra-class correlation coefficient were calculated. To examine validity, correlation and partial correlation analyses were conducted.

**Results:**

Results indicated that the factor structure of the Japanese AELS was equivalent to that of the original AELS (CFI = .942, TLI = .920, RMSEA = .055). The scale had adequate internal reliability (sensing: α = .64/ ω = .72, processing: α = .61/ ω = .62, responding: α = .68/ ω = .77, total: α = .82/ ω = .86) and a moderate test-retest intraclass correlation coefficient (sensing: .53, 95%Cl [.31–.69], processing: .48, 95%Cl [.26–.65], responding: .52, 95%Cl [.31–.68], total: .51, 95%Cl [.29–.68]). Criterion-related validity was demonstrated by the positive correlation between the Japanese AELS and other measures (IRI and ENDCOREs).

**Conclusions:**

These results suggest that the validity of the Japanese AELS is relatively comparable to the original AELS; however, it will be necessary to determine potential cultural concerns by comparing Japanese culture and other Asian cultures in future studies. The Japanese AELS is expected to be used to measure the outcomes of active listening training in Japan.

## Background

Active listening and empathic listening are related to psychologically supporting others [1, 2]. Recently, regarding the listening process, the concept of active empathic listening, which combines active listening and empathic listening, has been developed [3, 4]. Since active emphatic listening research has been mostly conducted in the United States, the purpose of the present study was to create and confirm the validity and reliability of a Japanese version of the Active-Empathic Listening Scale (AELS) for use in Japan, which is culturally different from the United States.

Active empathic listening focuses on empathy. Empathy is an important ability needed to maintain a society that inhibits aggressive behavior and promotes the support and understanding of others [5]. In psychotherapy, empathy is an important factor in many mental health counselling theories [6]. Although there are various definitions for empathy [7], for a psychotherapy setting, empathy has been defined as the essential therapeutic condition of being able ‘to sense the client’s private world as if it were your own’ [8]. In other words, it is important that therapists not only listen to their clients, but also show them empathy. Therefore, empathy has generally been considered to be closely associated with listening in therapeutic settings.

Active empathic listening includes active listening as well. Rogers and Farson [9] also call for ‘active listening,’ which encourages change in people by helping to create therapist-client relationships of understanding, acceptance, and warmth. Specifically, active listening includes demonstrating one is paying attention, conveying responsiveness and empathy, asking appropriate questions, paraphrasing and summarizing the speaker’s message, and using verbal and/or non-verbal communication to clearly convey interest to the speaker [1, 10]. Active listening is not only a special skill for therapists but also a useful skill for many people in their everyday lives, such as in informal helping conversations [1], as well as in educational [10], medical, [11] and occupational settings [12–14]. In one study, employees working under a manager with a high active listening score reported less psychological stress than those working under a manager with a low active listening score [13]. Furthermore, according to Kubota, Mishima, and Nagata [14], active listening is a skill that can be learned in a one-day training, and the positive effects of such training have been shown to persist even in a three-month follow-up.

As empathic listening includes active involvement in a conversation such as paraphrasing, asking questions, and summarizing [2], emphatic listening and active listening can be viewed as being related to each other. To measure active empathic listening, which combines active listening and empathic listening, the AELS was developed with the following three dimensions: ‘sensing,’ ‘processing,’ and ‘responding’ [3, 4]. ‘Sensing’ denotes the listener has paid active attention to the speaker’s emotions, as well as both the implicit and explicit aspects of the speaker’s message. ‘Processing’ indicates the listener has understood, interpreted, evaluated, and remembered contents of conversations and appropriately integrated the different parts. ‘Responding’ shows the listener has used verbal and nonverbal communication, such as asking relevant questions, to demonstrate active concern toward the speaker. The AELS is a measurement tool that was originally created to assess the communication skills of salespeople [4]. Subsequently, Bodie [3] adapted the original AELS for use within the general population. The AELS has been shown to have temporal stability [15] and is associated with social skills [16], biological sex [17], and intrapersonal communication in which individuals indirectly experience or imagine communication with others [18]. The AELS can be administered by other-report and behavioral coding as well as by self-report [3, 19]. Furthermore, the AELS has also been successfully translated into languages other than English, such as a Greek version which has been shown to have high reliability and validity for use with teachers [20, 21].

Therefore, the AELS assesses listening abilities, which include responses to the speaker such as verbal acknowledgements or body language that conveys attentiveness. The AELS is also understood as a form of showing social support toward others [19]. Since the AELS has been used most frequently in the United States [3, 17, 18], its reliability and validity have not been confirmed in cultures that use different communication styles than those common in the United States. This includes Japan, which is a high-context society, where more attention is paid to non-verbal communication than in a low-context society, such as that of the United States [22, 23]. Therefore, in Japan, there might need to focus more on the sensing factor of the AELS (e.g. ‘I am sensitive to what others are not saying’) than in a low-context society. Thus, developing a Japanese version of AELS would not only contribute to research on the AELS but also to research on communication and social support.

To verify the criterion-related validity of the Japanese AELS, we used the Interpersonal Reactivity Index (IRI) [24, 25] to assess empathy, and the Encode, Decode, Control, and Regulate Model (ENDCOREs) [26] to assess social skills. Drollinger et al. [4] argued that empathy is an essential component of all processes related to active empathic listening. Furthermore, Bodie [3] demonstrated a positive correlation between the empathic responsiveness scale [27] and the AELS. Therefore, we hypothesized that the AELS would be positive associated with IRI.

Regarding social skills, the three factors of the AELS were shown to be positively correlated with emotional express (EE), emotional sensitivity (ES), social expression (SE), and social control (SC) on the Social Skills Inventory (SSI) [28], and the sensing and responding factors of the AELS were further demonstrated to have a positive correlation with social sensitivity (SS) in a sample of American college students [16]. ENDCOREs, which was used to assess social skills in the present study, is the most popular model in Japan for measuring communication skills. Therefore, we hypothesized that each aspect of the AELS would be positively related to the corresponding aspects of ENDCOREs.

In this study, the factors mentioned above were examined to reveal whether they are related to the AELS. The aim of the present study was to demonstrate and report on the reliability and validity of the Japanese AELS.

## Methods

### Participants

**Fig. 1:**
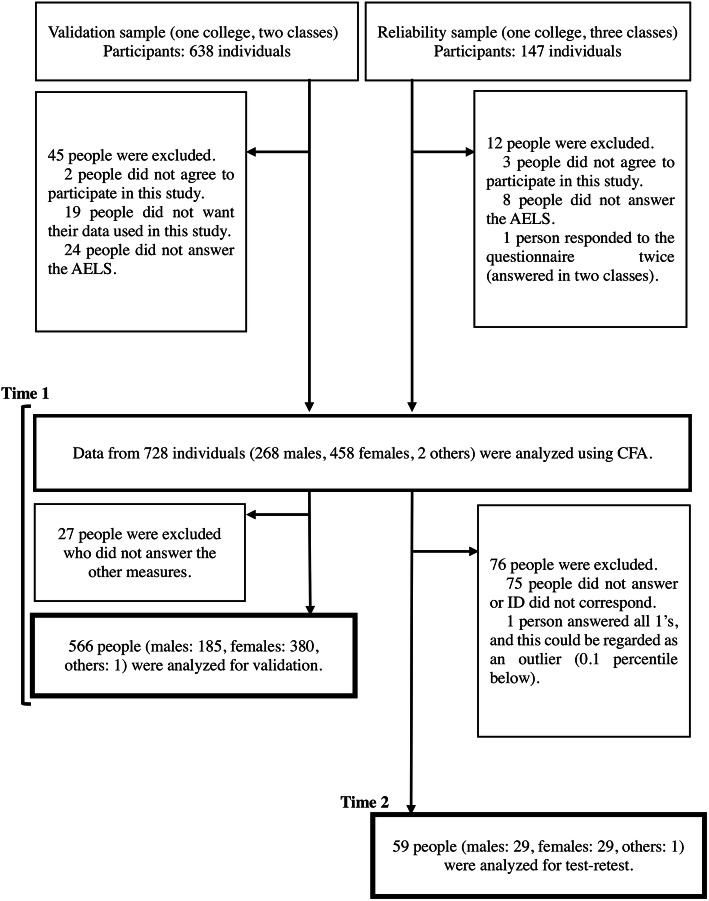
Participants’ flow diagram. Note: Time 1 was conducted for CFA (*n* = 728) and validation (*n* = 566). Time 2 was conducted for retest (*n* = 59). Time 2 occurred 3 weeks after Time 1

This study included 785 university students as volunteer participants. They were recruited from five classes at two different universities. The survey was conducted from April to July 2019. One group of participants was used as the validation sample (*n* = 638) and another group was used as the was retest sample (*n* = 147). After excluding 57 individuals who did not agree to participate in the study, did not want their data used, or responded to the same questionnaire in more than one class, data from a total of 728 individuals (268 males, 458 females, 2 others; *M* = 19.15 years, *SD* = 1.10) were analyzed. Of those participants, 566 individuals responded to other measures for validation and 59 individuals were included in the retest sample and re-took to the Japanese AELS (see Measures section) after 3 weeks. Figure 1 depicts the participants’ flow diagram.

### Measures

#### Japanese version of the AELS

Bodie [3] developed the AELS, which has 11 items rated on a 7-point Likert-type scale ranging from 1 (never or almost never true) to 7 (always or almost always true). The reliability of the adapted AELS was confirmed by its internal consistency (Cronbach’s α > .66) and the re-test method (*r* > .70), and its validity was confirmed by the criterion-related validity of both the active and empathic aspects [3, 15]. After obtaining permission from the scale’s original developer, Dr. Bodie, we translated the English version of the AELS into Japanese. We carefully followed the standard procedure of back-translation. The first author translated all items from English into Japanese (preliminary translation). This preliminary Japanese AELS was then back translated into English by an independent translator (back-translation). The original developer, Dr. Bodie, checked the back translated Japanese AELS and provided feedback to the first author. The first author then incorporated those comments into a final revision of the Japanese AELS.

#### Japanese version of the IRI

The IRI [24] is a 28-item self-report scale that measures dispositional empathy and comprises the following four subscales: ‘personal distress,’ ‘empathic concern,’ ‘perspective taking,’ and ‘fantasy scale.’ Personal distress represents the tendency to experience distress and discomfort when observing the distress of others. Empathic concern denotes the tendency to experience feelings of other-oriented emotions, such as sympathy and compassion. Perspective taking signifies the extent to which one considers the point of view and feelings of others. Fantasy scale represents the tendency to imagine oneself in the place of fictional characters. Himichi et al. [25] confirmed the validity of the Japanese version of the IRI. The 28 items are each rated on a five-point scale ranging from 1 (not true at all) to 5 (extremely true).

#### ENDCOREs

ENDCOREs is a 24-item self-report scale that measures a variety of verbal and non-verbal communication skills [26]. The scale uses a 7-point Likert-type scale ranging from 7 (very good at) to 1 (very bad at). This scale comprises six subscales, each measuring a different communication skill: self-control (e.g. I can control my impulses and desires), expressivity (e.g. I can express my thoughts through words well), decipherer ability (e.g. I can feel sensitive toward an opponent’s emotional and psychological state), assertiveness (e.g. I can advance the conversation by taking the initiative), other-acceptance (e.g. I can respect the opinions and positions of others), and regulation of interpersonal relationship (e.g. I can act to give human relationships top priority). Each subscale has four items.

These skills are measured using three systems: the encode system, which includes expressivity and assertiveness; the decode system which includes decipherer ability and other acceptance; and the management system, which encompasses self-control and regulation of interpersonal relationships. Additionally, expressivity, assertiveness, decipherer ability, other acceptance, regulation of interpersonal relationships, and self-control on ENDCOREs were shown to correspond to EE, SE, ES, SS, SC, and emotional control (EC) on the SSI, respectively [26].

### Procedures

This study was approved by the Ethics Committee of Hokkaido University of Education. The survey was administered to student participants during class time. Class attendees were informed of the study’s purpose, confidential methods for data processing used to protect anonymity, and that participation was optional and it would not affect them negatively if they chose not to participate. Individuals who expressed agreement in participating provided informed consent before completing the survey. Additionally, we asked participants for their permission to use their survey responses in our study. To maintain anonymity, participants in the test-retest were identified by using a unique-number combination (the last digit of his/her student number, birthdate, and the last two digit of his/her mobile phone number).

### Statistical analyses

Confirmatory factor analysis (CFA) was performed using the Mplus 8.1 statistical software package [29]. The robust maximum likelihood estimator (MLR) was applied for the CFA estimation in this study. Missing data were calculated using the process of full information maximum likelihood (FIML). To test goodness of fit, we conducted the following analyses: comparative fit index (CFI), Tucker-Lewis index (TLI), root mean square error of approximation (RMSEA), standardized root mean square residual (SRMR), Akaike’s information criterion (AIC), Bayesian information criterion (BIC), and Akaike’s Bayesian information criterion (ABIC). As additional analysis, multiple-group CFA was conducted to examine gender invariance. Measurement invariance was tested by creating three models: a configural model (no constraints), metric invariance model (with item loading constraints to be equal across groups), and scalar invariance model (with item loadings and item intercepts with simultaneous constraints to be equal across groups). Following the hierarchy of these nested models, they were compared to each other. We focused on Satorra-Bentler chi-square values and ΔCFI as indicators in model comparisons. Satorra-Bentler chi-square test values were adjusted for non-normality [30]. Non-significant Satorra-Bentler chi-square values and ΔCFI smaller than .01 indicate model invariance [30, 31].

Other analyses were conducted using the computer program R 3.6.0 [32]. Cronbach’s alpha coefficients, McDonald’s omega coefficients, and correlations between the Japanese AELS and other measures were established by calculating Pearson’s correlation coefficients. Cronbach’s alpha coefficient values that fall between .65 and .80 are considered acceptable [33]. In contrast to Cronbach’s alpha coefficient, average inter-item correlation (AIIC) is independent from the number of items and sample size. AIIC values should theoretically fall between .15 and .50. Values greater than .50 indicate redundant scale items, whereas values below .15 indicate that items are unrelated within a scale [34]. The test-retest correlation coefficient was calculated by using ICC (intra-class correlation coefficient, two-way random effects, and absolute agreement). Previous research has shown that when power is set to at least 80% and alphas and power are fixed at 0.05, a minimum sample size of 22 is sufficient to detect a value of 0.50 for the ICC; when permitting for an additional 20% drop-out rate, a sample size of 28 would be needed [35]. All statistical analyses used two-tailed tests. For all statistical evaluations, *p* values less than 0.05 were considered indicative of significant differences. Pairwise deletion was used for missing data.

## Results

### Factor structure

Mean scores for each item of the Japanese AELS are shown in Table 1. Mean values for each item ranged from 3.91 to 5.70, with standard deviations ranging from 1.32 to 1.70. Skewness and kurtosis were smaller than 1, except for items 8 and 11. Therefore, it was determined that items 8 and 11 showed negative distortion, whereas all other items showed a generally normal distribution. Moreover, the correlation coefficient between items 8 and 11 was more than .60. Confirmatory factor analyses, as described by Bodie [3], were used to examine the goodness of fit. The results showed that for the Japanese AELS, the second-order model did not fit well (Table 2, Model 1). To improve the model fit, modification indices (MIs) were used. Some of the constraints on the errors were released for modifying the model. The MI between items 8 and 11 (MI = 175.368) was the highest value. Therefore, a within factor error-covariance between items 8 and 11 was included, and the model was modified. As shown in Table 2, Model 2, the results indicated that the modified model was more acceptable (CFI = .942, TLI = .920, RMSEA = .055). This was the final model.

**Table 1:** Descriptive statistics of the Japanese AELS items

	Mean (SD)	Skewness	Kurtosis	Item 1	Item 2	Item 3	Item 4	Item 5	Item 6	Item 7	Item 8	Item 9	Item10	Item11
Item 1	4.25 (1.70)	−0.05	− 0.97	–	.37***	.12***	.38***	.22***	.21***	.20***	.19***	.24***	.22***	.14***
Item 2	4.47 (1.53)	−0.22	−0.69		–	.32***	.36***	.30***	.29***	.32***	.25***	.34***	.30***	.20***
Item 3	4.72 (1.32)	−0.50	0.01			–	.29***	.33***	.23***	.29***	.33***	.36***	.33***	.28***
Item 4	4.23 (1.48)	−0.10	−0.61				–	.29***	.34***	.40***	.28***	.35***	.32***	.33***
Item 5	4.04 (1.47)	0.05	−0.74					–	.29***	.31***	.30***	.37***	.36***	.27***
Item 6	3.91 (1.50)	0.04	−0.71						–	.42***	.16***	.35***	.33***	.14***
Item 7	4.79 (1.39)	−0.43	−0.41							–	.27***	.43***	.30***	.31***
Item 8	5.66 (1.34)	−1.20	1.29								–	.27***	.28***	.62***
Item 9	4.07 (1.40)	−0.00	−0.69									–	.41***	.26***
Item 10	4.48 (1.49)	−0.27	−0.60										–	.25***
Item 11	5.70 (1.35)	−1.24	1.27											–
Sensing	17.67 (4.20)	−0.16	0.05	.71***	.74***	.58***	.73***	.41***	.38***	.43***	.37***	.46***	.41***	.33***
Processing	12.73 (3.26)	−0.12	−0.22	.28***	.41***	.38***	.46***	.72***	.77***	.76***	.33***	.51***	.44***	.32***
Responding	19.90 (3.98)	−0.71	1.02	.28***	.38***	.46***	.45***	.46***	.35***	.46***	.74***	.68***	.70***	.73***
AELS	50.30 (9.60)	−0.44	0.78	.52***	.62***	.57***	.66***	.61***	.57***	.64***	.58***	.66***	.62***	.56***

All items *n* = 728, except for Item 3 (*n* = 727)

***p < .001

**Table 2:** Factor loadings and goodness of fit for the Japanese AELS

Items	Standardized loadings		
	Model 1	Model 2	α/ω
AELS total			.82/.86
Sensing	.925	.910	.64/.72
1 I am sensitive to what others are not saying.	.458	.462	
2 I am aware of what others imply but do not say.	.589	.596	
3 I understand how others feel.	.529	.526	
4 I listen for more than just the spoken words.	.644	.639	
Processing	.955	.970	.61/.62
5 I assure others that I will remember what they say.	.565	.565	
6 I summarize points of agreement and disagreement when appropriate.	.547	.556	
7 I keep track of points others make.	.645	.638	
Responding	.933	.998	.68/.77
8 I assure others that I am listening by using verbal acknowledgements.	.568	.459	
9 I assure others that I am receptive to their ideas.	.634	.657	
10 I ask questions that show my understanding of others’ positions.	.579	.584	
11 I show others that I am listening by my body language (e.g., head nods).	.552	.439	
Χ^2^	303.405***	128.980***	
*df*	41	40	
CFI	.828	.942	
TLI	.769	.920	
RMSEA	.094	.055	
90 Percent C.I.	.084–.104	.045–.066	
SRMR	.058	.040	
AIC	27,080.457	26,872.207	
BIC	27,245.707	27,042.048	
ABIC	27,131.396	26,924.562	

*CFI* Comparative fit index, *TLI* Tucker-Lewis index, *RMSEA* Root mean square error of approximation, *SRMR* Standardized root mean square residual, *AIC* Akaike’s information criterion, *BIC* Bayesian information criterion, and *ABIC* Akaike’s Bayesian information criterion

***p < .001

The configural invariance of the final model was acceptable, as indicated by the values of the fit indices when gender groups were estimated without constraints. Satorra-Bentler chi-square test did not reveal significant differences between the metric invariance and configural models ([Δχ2(11) = 10.25, *n.s.*] and ΔCFI = .004). The metric invariance and scalar invariance models showed significant Satorra-Bentler chi-square value ([Δχ2(11) = 112.39, *p* < .001] and ΔCFI > .01). Consequently, the metric invariance model was supported (Table 3).

**Table 3:** Measurement invariance of model 2 among gender groups for the Japanese AELS

	χ^2^	df	CFI	Δdf	Δχ^2^	ΔCFI
Configural model	188.716***	80	.930			
Metric invariance model	192.764***	91	.934	11	10.25	−.004
Scalar invariance model	270.872***	102	.891	11	112.39***	.043

*CFI* Comparative fit index

Δχ2 was calculated using Satorra-Bentler chi-square test

***p < .001

### Reliability and validity of AELS

The descriptive statistics of the Japanese AELS are presented in Table 4, which shows the intercorrelations between the domains were all significant, and ranged from *r* = .54 to *r* = .56. Internal consistency reliability was acceptable for sensing (α = .64/ ω = .72), processing (α = .61/ ω = .62), responding (α = .68/ ω = .77), and total AELS scores (α = .82/ ω = .86). Average inter-item correlations for the three subscales ranged from .31 to .35, whereas average item to nontarget total correlations for the three subscales ranged from .28 to .29.

**Table 4:** Descriptive statistics of the Japanese AELS

	Min-max	AIIC	Average item to nontarget total correlations	Processing	Responding
Sensing	4–28	.31	.28	.54***	.55***
Processing	3–21	.34	.29	–	.56***
Responding	4–28	.35	.28		–

Sensing *n* = 727; Processing *n* = 728; Responding *n* = 728

***p < .001

To examine relative validity, the correlations between each of the three domains of the AELS and other scales were calculated. The correlation coefficients are presented in Table 5. Regarding the IRI, the alpha coefficient for ‘perspective taking’ was below the acceptable value. Previous research on the IRI’s ‘perspective taking’ subscale (seven items) has been found to be less reliable, due to the inclusion of two reverse-scored items [25]. However, as research has also indicated there are no significant changes in CFA results or the correlations between each subscale when using all seven items including the reverse-scored items, compared to using only five items excluding the reverse-scored items [25], we used all seven items in the present study. The correlations between each domain for the Japanese AELS and the domains of the IRI were significant, ranging from *r* = .20 to *r* = .30, *p* < .01 (empathic concern) from *r* = .25 to *r* = .39, *p* < .01 (perspective taking), and from *r* = .20 to *r* = .28, *p* < .01 (fantasy scale). Personal distress showed a significantly negative correlation only for processing (*r* = −.10). Therefore, our hypothesis was supported for all factors expect personal distress. All domains of the Japanese AELS were significantly positively correlated with all communication skills from ENDCOREs (*r* = .16–.52). Especially, ‘decipherer ability’ was low and moderately positively correlated with all AELS aspects (*r* = .37–.52). Therefore, our hypothesis was supported. Partial correlations were used to examine unique relations between each AELS factor and the factors of the IRI and ENDCOREs, while controlling for other AELS factors. The results were generally similar to those of the correlation analysis: sensing was positively correlated with all IRI factors except personal distress and all ENDCOREs factors except expressivity (*r* = .08–.35); processing was weakly positively correlated with assertiveness of ENDCOREs (*r* = .20); responding was weakly positively correlated with empathic concern of IRI (*r* = .19) and other acceptance of ENDCOREs (*r* = .23).

**Table 5:** Correlations between the Japanese AELS and other scales

			AELS		
			Sensing	Processing	Responding
IRI	.78	.82			
Personal distress	.76	.79	−.06 (−.03)	−.10* (−.11*)	.01 (.09*)
Empathic concern	.73	.82	.23*** (.08*)	.20*** (.01)	.30*** (.19***)
Perspective taking	.56	.71	.39*** (.26***)	.33*** (.14***)	.25*** (.01)
Fantasy scale	.76	.81	.25*** (.11**)	.20*** (.01)	.28*** (.17***)
ENDCORES	.90	.92			
Self-control	.63	.70	.29*** (.14**)	.31*** (.17***)	.24*** (.04)
Expressivity	.72	.76	.16*** (−.02)	.26*** (.14***)	.27*** (.15***)
Decipherer Ability	.84	.85	.52*** (.35***)	.37*** (.05)	.42*** (.18***)
Assertiveness	.74	.77	.31*** (.13**)	.36*** (.20***)	.27*** (.04)
Other Acceptance	.73	.77	.37*** (.21***)	.26*** (−.02)	.39*** (.23***)
Regulation of Interpersonal Relationship .	.73	80	.29*** (.15***)	.26*** (.08*)	.25*** (.08*)

Parentheses are used for partial correlations, controlling for the other two variables. *N* = 566

*p < .05, ** *p* < .001, ***p < .001

Results for the test-retest reliability of the Japanese AELS (ICC; intra-class correlation coefficient, two-way random effects, absolute agreement) were as follows: sensing = .53 (*p* < .001; 95%Cl [.31–.69], *n* = 56; ICC for each item = .17–.55); processing = .48 (*p* < .001; 95%Cl [.26–.65], *n* = 59; ICC for each item = .35–.59); responding = .52 (*p* < .001; 95%Cl [.31–.68], *n* = 59; ICC for each item = .23–.51); and AELS total score = .51 (*p* < .001, 95%Cl [.29–.68], *n* = 56). ICC values were evaluated by using the following criteria: < 0.5 = poor, 0.5–0.75 = moderate, 0.75–0.9 = good, and > 0.90 = excellent [36]. Therefore, test-retest reliability of the Japanese AELS and each of its aspects were considered to be moderate, except in the case of processing.

## Discussion

This study developed the Japanese AELS. Factor and reliability analyses confirmed the three-dimensionality of the scale for the aspects of ‘sensing,’ ‘processing,’ and ‘responding.’ As expected, the Japanese AELS was positively correlated with the IRI and the ENDCOREs. The results indicate that the Japanese AELS has acceptable reliability and validity. Furthermore, these findings reveal that the Japanese AELS can be regarded to have a similar three-dimensional higher-order construct in common with the version of the AELS developed by Bodie [3].

In factor structure, when assuming an error correlation, goodness of fit becomes an acceptable value. Our findings suggested that our participants regarded item 8 (‘I assure others that I am listening by using verbal acknowledgements’) and item 11 (‘I show others that I am listening by my body language [e.g. head nods]’) to be different from the other items. From the results of each item correlation, these two items also showed a moderate correlation to each other when compared to other items on the scale. Japan has a highly contextual culture, which focuses more on non-verbal cues than verbal cues [22, 23]. Moreover, previous studies have suggested that Japan may have a more high-context culture than China, which is another Eastern country, because, historically, Japanese people have interacted less with other ethnic groups than Chinese people, and the Japanese language is characterized by the use of implied meaning not only in everyday conversation but also in formal communication [23]. Therefore, this aspect of Japanese culture could explain the high correlation between items 8 and 11. Therefore, it is especially necessary to clarify whether the correlation between items 8 and 11 is a phenomenon specific to Japan, or if it would be found in other highly-contextual cultures as well. To clarify this issue, a comparative study of the AELS for different Asian cultures should be conducted in the future.

Multiple-group CFA by gender was conducted. As a result, similar factor structures and factor loadings were found for both men and women, and AELS was shown to be able to measure active empathic listening on three subscales for both men and women. However, a gender comparison using the AELS scores were not recommended because it was unclear whether the latent factor variances had the same metric across genders. In empathy studies, it has been shown that women are better at emotion management, recognition, and prosocial behavior [37]. These gender differences in empathy may have influenced the unsupported scalar invariance model of the AELS.

Cronbach’s alpha coefficients for the AELS did not show high values. However, reliability of the AELS was considered to be acceptable, since AIIC values, which are less affected by the number of items on a scale, met acceptable criteria. AIIC values were larger than average item to nontarget total correlations. Further, reliability was low in ‘processing’ from the viewpoint of internal consistency and test-retest reliability. This finding was similar to other previously observed phenomena (study 1, α = .66 [3]; α = .66 [16]; α = .67 [18]). This could indicate that ‘processing’ is not compatible with Japanese culture and/or ‘processing’ is not a good factor on the AELS. ‘Sensing’ and ‘responding’ showed more emotional responses than ‘processing’ [3]. Additionally, Gearhart and Bodie [16] pointed out the possibility that active-empathic listening has more to do with ‘sensing’ and ‘responding’ than ‘processing’. Therefore, perhaps the processing factor of the AELS may be more dependent on the conversation than the other factors. Bodie et al. [15] showed that participants with high AELS scores consistently had high scores regardless of which of the study’s four scenarios was used. However, these scenarios all depicted a one-on-one interaction with a close friend or partner and were presented in less than 100 words; therefore, situations where one would be required to understand or integrate complex content or information into a conversation were not included by Bodie et al. [15]. Thus, the processing factor of the AELS may be better realized in scenarios presenting conversations that allow for complex information processing, such as ones in which a wide variety of topics are discussed or that involve several people. The AELS instructs individuals to ‘please read each statement and indicate how frequently you perceive it is true about you using the following scale,’ and does not specify a situation. To clarify this point, future studies are required to confirm that AELS scores, especially for processing, are stable, and would not change depending on the number of people in or topics of a conversation. In addition, the combined score for the Japanese AELS and the other two factors (‘sensing’ and ‘responding’) also did not have good test-retest reliability. Since Japan is a high-context society, it is likely that required listening skills are situation-dependent. Thus, it is possible that the situations envisioned by respondents in the first and second surveys may have differed. This may be clarified by asking participants to respond to the AELS multiple times, with instructions on various conversation situations. Bodie et al. [15] evaluated the retest reliability of the AELS using a correlation coefficient instead of ICC. ICC is more appropriate than Pearson’s correlations in test-retest situations, because Pearson’s r will not necessarily accurately evaluate test-retest reliability in all situations, such as, for example, when retest scores are uniformly higher than the original test scores [38]. Thus, further research is required to reveal whether only the Japanese version of the AELS has a moderate ICC, or if the AELS overall has a moderate ICC.

We considered that the criterion validity of the Japanese AELS was met due to the each AELS factor being positively correlated with each IRI factor except personal distress, and each AELS factor being positively correlated with ENDCOREs. Results of the partial correlation analysis revealed the characteristics for each factor: ‘processing’ correlated with assertiveness of ENDCOREs and had the characteristic of active attitude; ‘responding’ correlated with empathic concern of the IRI and had the characteristic of empathy; ‘sensing’ correlated with each aspect of ENDCOREs, except expressivity, compared to the other two factors, which may measure the basic ability to understand what another person is saying. Thus, the three factors have different characteristics, which may be an appropriate measure of active emphatic listening. However, both in correlation analysis and in partial correlation analysis, the reason why personal distress does not have a weak positive correlation with the AELS is possibly because personal distress, which measures the occurrence for non-daily emotional pain, is significantly different from the everyday communication measured by the AELS. Regarding communication skills, our findings showed a positive correlation with all ENDCOREs factors, which is not in line with the findings of Gearhart and Bodie [16]. We considered this finding to be related to the desirable attitude of interpersonal relationships in Japan. Over-adaptation, which indicates a tendency for individuals’ thoughts and behaviors to follow the expectations of others as perfectly as possible, even if their own desires and emotions are unduly repressed, is not uncommon in Japan [39, 40]. Although over-adaptive people might seem like they have adapted well, they tend to suffer from psychosomatic disorders, psychoneurosis, and/or school refusal [41]. Thus, since self-inhibition is considered an adaptive behavior in Japan, all ENDCOREs factors, including ‘self-control,’ were positively correlated with each aspect of the Japanese AELS. Furthermore, the Japanese AELS was able to capture not only the ‘listening’ aspect but also the ‘active’ aspect, due to a positive correlation with the encode (expressive and assertive) and management (self-control and regulation of interpersonal relationships) systems. Based on these findings, the Japanese AELS can be said to have a second-order factor structure similar to the original version of the AELS [3].

The present study has some limitations. As mentioned above, one of the major limitations is it did not demonstrate good test-retest reliability. Further studies will be needed to determine if the moderate reliability is caused by cultural differences or the small sample population. In the future, it will be necessary to compare Japanese culture to other cultures, increase the population sample for test-retest, and prolong the period for test-retest. Additionally, the present study was a translation of the self-report version of the AELS. As Bodie [3] also created an other-report version of the AELS, it will also be necessary for future studies to provide a Japanese translation of the other-report version and compare the self-report and other-report Japanese versions of the AELS. Furthermore, the study’s participants were Japanese university students. In the case of university students, there are likely to be occasions in lectures and other aspects of university life where they are required to summarize the points of, or otherwise show they understood, another person’s story. However, in the general population, it is possible that the frequency of situations that require active empathic listening could differ by occupation. Active empathic listening may be required on a daily basis for service industry workers, such as salespeople, and human service professionals, such as teachers and counsellors; however, it may not be necessary to use active empathic listening in occupations that do not emphasize interpersonal communication. In the future, it will be necessary to investigate whether the same factor structure as found in the present study can be replicated for different occupations, in addition to surveying a sample of non-university students.

## Conclusions

Despite these limitations, this study demonstrates that the Japanese AELS has good internal consistency and moderate test-retest reliability. A positive correlation among the AELS, IRI, and ENDOCOREs in this study suggests that the validity of the Japanese AELS might be relatively comparable to the original AELS. Regardless of cultural differences, empathy is required to engage positively in social situations. Because active empathic listening is a skill that shows empathy, it is a skill important for all people, not only mental health professionals. The Japanese AELS is expected to be used for people to assess their own active empathic listening abilities and to measure the outcomes of active empathic listening trainings conducted in Japan. Moreover, research using this scale is expected to contribute to communication and social support research in Asian countries, including Japan. Ultimately, using active empathic listening provides an opportunity to strengthen interpersonal relationships and avoid confrontational communication styles, which could contribute to individual well-being and/or positive mental health outcomes.

## Data Availability

The datasets used and/or analyzed during the current study are available from the corresponding author on reasonable request.

## Abbreviations

ABIC: Akaike’s Bayesian information criterion
AELS: Active-Empathic Listening Scale
AIC: Akaike’s information criterion
AIIC: Average inter-item correlation
BIC: Bayesian information criterion
CFA: Confirmatory factor analysis
CFI: Comparative fit index
EC: Emotional control
EE: Emotional express
ENDCOREs: Encode, Decode, Control, and Regulate Model
ES: Emotional sensitivity
FIML: Full information maximum likelihood
ICC: Intra-class correlation coefficient
IRI: Interpersonal Reactivity Index
MIs: Modification indices
RMSEA: Root mean square error of approximation
SC: Social control
SE: Social expression
SRMR: Standardized root mean square residual
SS: Social sensitivity
SSI: Social Skills Inventory
TLI: Tucker-Lewis index
